# Claw Trimming as a Lameness Management Practice and the Association with Welfare and Production in Dairy Cows

**DOI:** 10.3390/ani10091515

**Published:** 2020-08-27

**Authors:** Mohammed Babatunde Sadiq, Siti Zubaidah Ramanoon, Rozaihan Mansor, Sharifah Salmah Syed-Hussain, Wan Mastura Shaik Mossadeq

**Affiliations:** 1 Department of Farm and Exotic Animal Medicine and Surgery, Faculty of Veterinary Medicine, Universiti Putra Malaysia, UPM Serdang 43400, Malaysia; sadiquemohammed99@yahoo.com (M.B.S.); rozaihan@upm.edu.my (R.M.); 2 Centre of Excellence (Ruminant), Faculty of Veterinary Medicine, Universiti Putra Malaysia, UPM Serdang 43400, Malaysia; wmastura@upm.edu.my; 3 Department of Veterinary Clinical Studies, Faculty of Veterinary Medicine, Universiti Putra Malaysia, UPM Serdang 43400, Malaysia; ssalmah@upm.edu.my; 4 Department of Veterinary Pre-Clinical Sciences, Faculty of Veterinary Medicine, Universiti Putra Malaysia, UPM Serdang 43400, Malaysia

**Keywords:** dairy cows, claw trimming, claw lesion, lameness, animal welfare

## Abstract

**Simple Summary:**

Lameness impacts negatively on dairy cattle welfare and production. Claw trimming is a routine practice for lameness management in dairy cows. Claw trimming is often applied for the treatment of clinically lame cows especially those affected with horn lesions; however, the benefits in the area of prevention are not well understood. This issue results from a combination of paucity of data on preventive trimming and shortfalls in study designs, which limits our understanding of identifying cows that will benefit more from such intervention during lactation. Computerized claw trimming database programs have the potential of curtailing some of these issues, by providing the basis for monitoring hoof health and adjusting lameness management practices in dairy herds. This review summarizes literature findings regarding claw trimming methods, their application in lameness management, as well as associations with the welfare and production of dairy cows.

**Abstract:**

Lameness resulting from claw lesions remains a pressing welfare issue in dairy cows. Claw trimming (CT) is a common practice for prevention and management of clinically lame cows. This review summarizes the results of studies that have investigated various claw trimming (CT) methods, their application in lameness management, and associations with the welfare and production of dairy cows. The papers included in this review fulfilled the following inclusion criteria: published in peer review journal or book chapter within the last 20 years (1999–2019), written in English, and focused on the application of CT for lameness management and the association with either welfare or production variables. Databases used included Google scholar, Web of Science and PubMed. A total of 748 records were assessed and 61 papers were eligible for inclusion and the main objectives and results were used to categorize the results under six topics: CT techniques, association between CT and claw overgrowth/specific claw lesions, timing and frequency of CT, association between CT and behavioral variables, association between CT and physiological parameters, and association between CT and production. The literature findings showed the existence of various CT methods with the common types including the Dutch Five-step, White Line, White Line Atlas, and Kansas techniques. There is data paucity on the efficacy of these techniques in lameness management; however, the slight procedural difference yields varying sole thicknesses and presentations which may influence their prophylactic use. Results regarding the impact of CT on welfare and production were discussed in relation to potential short and long-term benefits. Depending on the lesion type and severity level, CT may induce immediate painful sensation, stress, changes in lying down activities and reduction in milk yield, but the positive impacts were more evident at later stages of lactation following improvement in locomotion score. The majority of the reviewed studies were lacking a detailed description of CT techniques and claw health of the studied animals; thus, reducing the strength of demonstrating CT-related benefits. However, electronic recording of claw health data during every CT visit provides the basis for monitoring hoof health and could assist in curtailing some of these challenges. To elucidate CT-related benefits, certain areas requiring further research were highlighted such as ascertaining the appropriate timing for preventive CT and identifying cows that will benefit more from such intervention during lactation.

## 1. Introduction

Dairy cow welfare is an important aspect in milk production systems [1,2]. Lameness impacts negatively on animal welfare [3] and remains a financial burden to dairy farmers [4]. Lame cows are in pain [5,6] and the adverse effects on behavioral variables such as lying down [1,3] and feeding activities [3], makes it the most pressing welfare issue. The related economic losses are accrued from reduced milk yield, poor reproductive performance, and shortened productive years [7]. 

Correct claw trimming (CT) by well-educated hoof trimmers or veterinarians is an essential lameness management procedure [8,9]. CT is aimed at ensuring better claw health by providing appropriate weight distribution between the medial and lateral claws [10,11]. There are various CT methods in the scientific literature with the major types including the functional trimming or Dutch method [12], White line method [13], White Line Atlas [14], and Kansas methods [15]. Though the procedures in each method are similar, the approaches yield measurable differences in sole thickness and presentation, which may influence their prophylactic or therapeutic purpose. However, their efficacies in lameness management have not been fully elucidated. 

Maintenance or preventive CT is primarily conducted for the management of claw disorders such as claw overgrowth and unbalanced sole or heel [16]. Preventive CT has been advocated by a limited number of research findings based on overall reduction in lameness incidence or lesion prevalence during lactation [17,18,19]. However, attributing such benefits solely to CT is limited due to factors inherent in study designs and failure to consider the claw health and lameness history of study population prior to CT. On the other hand, claw horn disruptive lesions (CHDL) are managed by corrective trimming in conjunction with other treatment protocols involving pressure reduction on the affected claw and pain management [20]. Corrective CT is also applied for the management of infectious claw lesions such as digital dermatitis (DD), where the interdigital cleft is adjusted to reduce exposure to manure slurry [21,22]. Hence, a review of the associations between CT and various claw lesions is important in understanding CT-related benefits in lameness management. 

Aside from claw health, CT also influences the behavior, physiological parameters, and milk yield; thus, culminating in an impact on welfare and production [5,23,24,25]. For instance, corrective CT induced immediate painful sensation [24] and longer lying down time in lame cows [25], while non-lame cows had increased cortisol levels [24,26] and reduced milk yield following functional CT [23]. Nevertheless, these associations with respect to the short-term and long-term effects are not well understood. 

Presently, there are no data that summarize the findings and extent of the research that has been conducted regarding the application of CT for lameness management, and the association with various welfare and production indicators in dairy cows. One of the means of identifying knowledge gaps and providing direction for future research is by conducting a systematic review of the literature [27]. The objective of this review is to assess the available CT techniques, application of CT for lameness and claw health management, as well as their association with welfare and production variables in dairy cows.

## 2. Materials and Methods 

**Figure 1:**
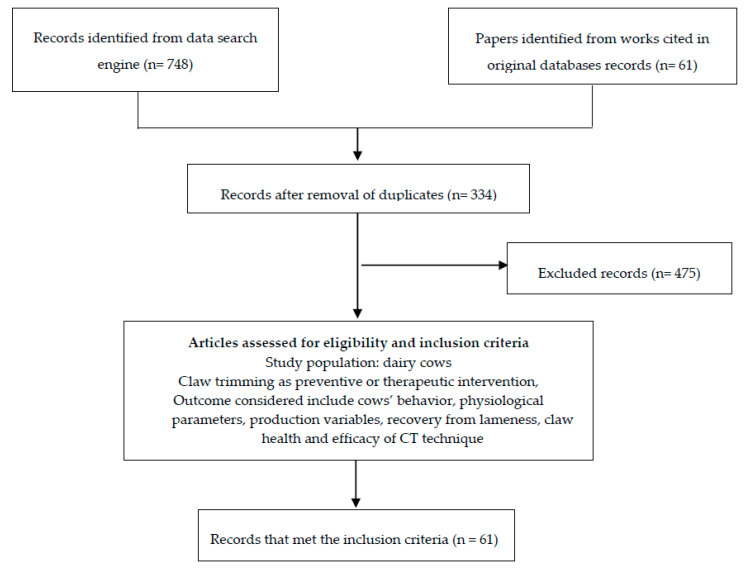
Flow diagram of study selection procedure.

The basic aspects of a systematic review were applied in this review. For papers to be included, they needed to fulfil the following criteria: published in peer review journal or book chapter, written in English and published between 1999 to 2019, focused on the application of CT for lameness management, and investigated the relationship between CT and welfare (behavioral and physiological parameters) and production variables (milk yield, reproduction performance or culling risk). The review was conducted from August 2019 to February 2020 and databases used for the literature search included Google scholar, Web of Science and PubMed. Additionally, the works cited in the papers obtained from the databases were evaluated for inclusion. The search terms used were “claw trimming”, “dairy cows”, “welfare”, “production”, “claw lesions”, and “lameness”. The 3 aforementioned databases yielded 748 records, whereas 61 cited papers were assessed from the original papers. The Preferred Reporting Items for Systematic Reviews and Meta-analyses (PRISMA) checklist was used for the literature selection process (Figure 1). 

Of the 809 records, 481 were identified as duplicates and they were excluded; thus, leaving 339 records for further review. After re-evaluation, 61 papers were eligible for inclusion and the objectives and major findings were used to classify the results under six main topics: CT techniques, association between CT and claw overgrowth/specific claw lesions, timing and frequency of CT, application of computerized CT database programs, association between CT and behavioral variables, association between CT and physiological parameters, and association between CT and production.

## 3. CT Techniques

The objectives of CT are to ensure both claws of the foot bear near to equal amount of weight and to preserve their function under various management systems. Results from the literature search yielded four major CT methods which comprised of the Dutch five step [12], White Line [13], White Line Atlas [14] and the Kansas method [15]. These CT techniques were categorized based on how the procedure presents the sole angle relative to the metatarsals. Hence, the functional and White line method were grouped under techniques yielding a relatively lower axial slope compared to the Kansas method. The White Line Atlas method is discussed separately. 

### 3.1. The Dutch Five-Step and White Line Method

The CT method developed by Toussaint Raven [12] is also referred to as the ‘Dutch five-step method’ or functional CT. The method advocates for relatively levelled abaxial and axial walls of the claw and they are presented perpendicular to the metatarsals. The initial step involves reducing the claw length, followed by trimming the thickest claw and establishing symmetry between the medial and lateral claws. Next, a dish is made at the axial wall, so as to minimize the local pressure at the solar region where sole ulcer (SU) develops [28]. The CT technique is widely applied in the dairy industry. As depicted in this review, the functional CT method was used in all the studies (*n* = 15) that provided a clear description of the applied CT. The White Line method proposed by Blowey [13] is based on a similar CT principle as found in the Dutch five-step method. A major step in the technique is examining the sole thickness during CT (sole reading) until the white line becomes visible at the toe region. 

The determination of sole thickness in both methods is critical to ensure that weight bearing between the lateral and medial hind claws is equal [29,30]. Based on the subsequent study conducted by Nuss and Paulus [31], a sole horn thickness of 7–8 mm is recommended so that upon levelling both hind claws, a sole horn thickness of at least 5 mm could be achieved on the lateral claw. Anatomical reasons account for the difference in the sole horn thickness between the medial and lateral claw, because the bones of the lateral digit are about 2–3 mm longer compared with that of the medial digit [31]. In both methods, the average toe length for an adult Holstein cow ranged from 3.00 to 3.25 inches. Heel height was not mentioned in the White Line method, but the functional method recommends an average of 1.5 inches [13]. 

### 3.2. White Line Atlas Method

The White Line atlas method was described by Vic Daniel and Randall White [14]. The basic principles in the CT technique is to ensure a trimming that allows for a unified bio-mechanical profile on each claw. Four major landmarks or biomarkers are employed to ensure proper execution of the CT, and they include identifying the white line or pressure line, normal sole thickness, claw length/claw angle and heel fulcrum. However, the method has not been described in a scientific publication nor has it been peer reviewed. 

### 3.3. Kansas Method 

This technique was developed based on the variations in hoof structure between animals [15]. Kansas method uses four characteristics namely wall length, heel depth, sole thickness and sole gradient to describe a normal hoof structure. These characteristics are used to ensure an objective definition of sole thickness and normal toe structure. Sole thickness is determined by the degree of dehydrated and shedding of overgrown horn [15]. In addition, the sole is trimmed to achieve average normal gradient and a slope sole. Such provision results in the axial wall (inner wall) being lower than the abaxial wall (outer wall) and presentation of a natural sole angle [15].

## 4. Association between CT and Claw Health 

### 4.1. Claw Overgrowth 

The dorsal wall length, dorsal wall angle and heel height are claw dimensions used to determine the presence of overgrown claws [9,16,29,31]. Claw overgrowth results when horn production occurs at a faster rate than the wear, with the majority of cases occurring on the hind limb claws. Overgrown claws resulting from disproportionate heel height are common in loose housing systems [18], whereas a too long dorsal wall is more prevalent in cows housed in tie-stalls [7].

Authors of the Dutch and White Line methods recommended an average dorsal wall length of 75 mm for a normal Holstein Friesian cow. However, while considering the influence of age and specific claw (medial and lateral), Nuss and Paulus [31] reported slightly different estimates for the lateral and medial claws, which were 75.6 and 76.3 mm for younger cows, and 78.0 and 77.8 mm for adult cows, respectively. The measurement point was from the proximal aspect of the perioplic horn (coronet), 1 cm abaxial to the interdigital space, to the distal end of the dorsal wall. Archer et al. [9] used the junction between the hoof horn and the adjacent integument as the proximal limit for measuring the dorsal wall length, and recommended a minimum length of 90 mm for adult Holstein cows. Hence, the definitions of the landmarks for the proximal dorsal wall limit are important and may influence the outcome of maintenance or therapeutic CT. 

Claw overgrowth is one of the main reasons for CT since it affects the weight bearing within and between the claws [11,30,31,32]. This disparity in weight bearing is profound on the lateral claw of the hind feet [31]. The functional CT method is to correct claw overgrowth and alterations in weight bearing by reducing the dorsal wall length and creating uniformity in sole thickness on each claw [28]. Functional trimming was found to reduce lameness prevalence in dairy cows [21] and the result was linked to benefits such as improvement in weight distribution, frictional properties at the floor–claw interface and claw dimensions and achievement of proper claw length and sole thickness [30,31,32,33]. Farms conducting routine CT only when cows were observed to have overgrown claws had higher levels of lameness [34]. In contrast, Dembele et al. [35] observed no significant difference in the prevalence of lameness and overgrown claws between farms that practiced and those that did not practice continuous trimming. Likewise, the number of months that elapsed before whole herd trimming had no influence on the prevalence of overgrown claws and lameness [35]. The fact that these studies were cross-sectional limits the generalization of the findings, while factors such as seasonal variation and housing designs could influence horn growth and the impact of CT [20]. 

### 4.2. Claw Horn Disruptive Lesions (CHDL) 

CHDL is a general term for lesions that arise from pathologies of the horn tissues. Examples of these lesions include sole ulcer (SU), sole hemorrhage (SH), white line disease (WLD), and toe ulcers (TU). CHDL are laminitis-related lesions following inflammation that sequel to breakage of the laminar corium from the hoof wall [36,37]. Recent findings have shown that the main impact for the development of these lesions is the overloading on the softer regions of the lateral hind claws, as the heel height of the lateral claw is higher than the medial claw (disproportionate heel height [16,28,38] and interplay between metabolic and hormonal changes due to energy deficiency during early lactation [39,40]. For instance, body condition loss, thinning of the digital cushion and weakening of the suspensory apparatus occurring post-calving reduces the load dissipation capacity of the sole soft tissues [39,40,41]; thus, increasing the likelihood of compressional corium injuries [37,38]. These events are further augmented by the lack of CT practices, improper trimming (failure to balance the medial and lateral claws) and long intervals between trimmings [31,32].

Corrective CT is carried out to relieve the pain associated with CHDL, which entails the removal of affected horn while preserving the healthy parts [5,42]. A modified functional trimming method focused on the achievement of correct claw angles and weight bearing reduced the risk of SH, SU, double sole and WLD during lactation [18,43]. The application of corrective CT for CHDL led to significant decrease in the proportion of lame cows [44] and those with poor gait (locomotion score; LS < 3) [45,46]. Several randomized clinical trials have demonstrated the benefit of early detection and treatment of CHDL in dairy cows [20,47,48]. Accordingly, the treatment consisting of corrective CT, 3-day course of non-steroidal and anti-inflammatory drug -ketoprofen, and block on the sound claw led to significant higher recovery rate (56%) compared to those that received only CT and 3-day course of ketoprofen (35.9%), CT and hoof block (29%) and untreated (24%) groups [20]. Such a positive impact was lacking when a similar treatment protocol was conducted in chronically lame cows (more than 2 weeks) affected with CHDL [42]. This reinstates that the effect of corrective CT on lame cows could be influenced by the type, severity and frequency of the CHDL [48]. 

For preventive CT, the incidence of CHDL was lower in farms conducting preventive CT compared to herds lacking the practice [20], while late lactation trimming reduced the likelihood of sole ulcers in subsequent lactation [49]. These studies suggested that preventive CT is beneficial in reducing horn lesions; however, attributing the results to CT is limited by the nature of the study design and lack of information on previous lameness and lesion history of the enrolled cows. 

### 4.3. Digital Dermatitis 

Infectious claw lesions affecting dairy cows include digital dermatitis (DD), interdigital dermatitis, heel horn erosion (slurry heel) and interdigital phlegmon (foot rot) [50]. Associations between functional CT and DD prevalence have been reported in few studies [51,52,53]. Long intervals between trimmings were associated with higher DD prevalence [52,53], while Holzhauer et al. [54] found that cows trimmed a year before they conducted their study had higher DD prevalence compared to those trimmed at shorter intervals. Aside being cross-sectional design studies, the disparity between the results is not fully understood due to paucity of data on the reasons for applying CT (maintenance or corrective). By using a longitudinal study design approach, the impact of preventive CT on DD occurrence can be elucidated; however, available data suggest that preventive CT alone has little effect on the prevalence of DD in dairy herds. For instance, preventive CT on farms endemic for DD led to lower CHDL prevalence, with no significant impact on infectious claw lesions [55]. 

The influence of therapeutic CT on recovery of DD affected cows has been demonstrated by some authors [22,56]. Manske et al. [56] reported significant reduction in DD lesions following treatment with glutarldehyde and corrective CT. The combination of allyl isothiocyanate (AIC; an oil-based organosulpur compound) and corrective CT resulted in significantly lower proportions of *Treponema*-like spirochetes (causative bacteria of DD) on the surface lesions compared to the group that only received AIC [22]. Aside from the effect of the chemical agent, the reduction in DD infection could be related to the application of CT in restoring a proper claw angle and heel height [18]. Such presentation transforms the heel area and reduces the exposure to slurry; thus, making the site unfavorable for the persistence of DD lesions [18]. Nevertheless, therapeutic trimming of DD affected cows needs to be applied with caution as contaminated hoof knives could serve as a means of cow-to-cow transmission [57]. This might have contributed to the higher DD prevalence in herds with shorter intervals between trimmings, as well as the visits to multiple farms by the same hoof trimmer [54,57]. These findings suggest that CT alone might be necessary but insufficient either for the prevention of DD or for the treatment of affected cows. Strategies to curtail cow-to-cow transmission and the persistence DD lesions are of utmost importance [58]. 

### 4.4. Other Claw Lesions

Aside from the lesions that have been documented typically as CHDL or infectious types, other important lesions include thin soles (TS), corkscrew claws (CSC), wall fissures, scissors claws, and interdigital hyperplasia (HYP) [50]. Amongst these claw lesions, thin soles are the lesions that have been consistently associated with CT practices in dairy herds. Thin soles (TS) are characterized by relatively lower sole horn thickness than the recommended threshold (≥5 mm [12], 7–8 mm [31]). Besides causing lameness, TS may also enhance the development of CHDL such as white-line disease and toe ulcers, thus resulting in severe pain and discomfort [59,60]. Mason et al. [60] recommended a cut-off measurement of ≤4.5 mm for TS in first parity Holstein-Friesian cows. Under such conditions, the horn growth rate is less than the horn wear rate [31,59]. 

The two major factors associated with occurrence of TS in dairy herds are incorrect trimming (over-trimming) and biomechanical events at the floor–claw interface [59,60,61,62]. Although the risk of over-trimming is present across lactation, the risk was suggested to be higher during 100–120 days in milk (DIM) as the lowest toe length was recorded during the period [63]. Based on the reports by Nuss and Paulus [31], strict adherence to the functional HT method (levelled lateral claws) led to significantly thinner soles. A recent study by Fuhrer et al. [62] found significant difference in the prevalence of TS in cows trimmed (at least 5 months before conducting study) and housed either on fully-floored mastic asphalt (53%) compared to those on partially-floored mastic asphalt (12.5%).

There is paucity of data on the association between CT and wall fissures and scissors claws, but there are few reports on HYP and CSC. Schulz et al. [47] reported that corrective CT resulted in lower prevalence of HYP during a 41 week trial, while the concurrent presence of DD and HYP in lactating cows during hoof examination was suggestive of positive association between the two lesions [64]. Infectious process has been implicated in the pathogenesis of HYP [64], but there is no current information on how corrective or preventive CT for DD or other infectious claw lesions might influence the levels of HYP. On the other hand, CSC is characterized by an inward curvature of the abaxial wall and can be managed by corrective CT [65]. However, there are no data to support the application of CT in preventing the lesion occurrence. This could be attributed to its hereditary nature [66,67] and relatively lower prevalence compared to other lesions in dairy herds [65,68]. 

**Figure 2:**
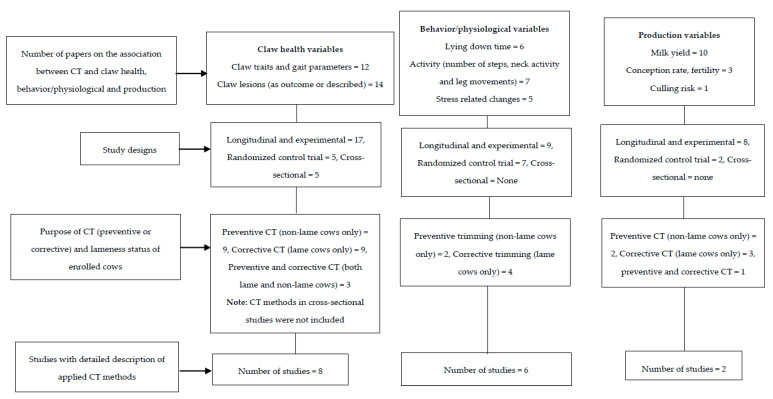
Summary of reviewed papers reporting the association between claw trimming and/or claw health, physiological, behavioral, and production variables in dairy cows, with information on study designs, rationale for and description of CT methods. The total number of studies in each of the uppermost boxes (claw health, behavior/physiological, production variables) could be greater than the numbers provided in the respective study designs, since some papers were counted more than once.

Overall, literature findings suggest that CT is important for the prevention and treatment of claw lesions. Despite the existence of various CT methods, there are few data on their comparative effects for lameness prevention in dairy herds [69]. Presently, there is only one published study on the impact of CT methods on lameness occurrence [28]. The authors found no significant difference in lameness incidence, LS, and claw conformation between the cows trimmed using the two CT methods. The common limitations in the reviewed papers are undetailed description of the applied CT technique, small sample size, short follow-up periods, and potential effects of cow-level confounders. Amongst the 21 reviewed papers involving either longitudinal or experimental designs, only 8 of them described the CT method and the basis for their usage (Figure 2).

## 5. Timing and Frequency of Corrective and Preventive CT 

Early detection and treatment of lame cows has been advocated by several research findings [5,20,48,70]. Corrective trimming led to lower lameness prevalence and LS at the first and fourth week post-CT, respectively, compared to the untrimmed groups [5,44]. Low recovery rates (15–16%) were observed in cows correctively trimmed 2 weeks after lameness detection [42]. For chronically lame cows, more than one corrective CT may be required in conjunction with other treatment protocols to improve the LS. This has not been investigated in the literature, but repeated treatments were conducted to reduce the incidence and recovery of cows affected with CHDL [42,44]. 

Preventive CT is aimed at reducing the likelihood of lameness occurrence during the high-risk period [17,71,72]. The highest frequency of lameness was between the second and fifth month of lactation, indicating that preventive claw trimming at dry off and again at DIM 40–60 could reduce the lameness prevalence during this period [73]. Application of CT during this period is to model the claw’s contact surface, thus, reducing the likelihood of corium injury resulting from instability of the pedal bone and weakening of the suspensory apparatus around calving. 

Cumulative incidence of lameness was lower (18%) in cows trimmed at mid-lactation compared to control group (24%) [21]. Recent reports suggest that trimming cows before dry-off resulted in lower odds of lameness [74,75] and reduced risk of sole ulcers in the subsequent lactation [49,71]. It is expected that the effect of late lactation/before dry-off CT will persist during early stages of the next lactation, however, these findings could not be exclusively attributed to CT since absence of claw lesion was not considered before cows’ enrollment [19,75] while the study by Thomsen et al. [49] was based on analysis of large CT records. Moreover, the benefits of CT at dry-off differed for cows at various parities, with results indicating more benefits for primiparous compared to multiparous animals [75]. 

Mahendran et al. [17] reported no significant difference in lameness episodes between dairy heifers trimmed at pre-calving, early lactation and control groups. Aside from low lameness levels in heifers, the differences in the environmental and management factors in the studied farms might have influenced the outcome. A recent cross-sectional study found low lameness levels amongst farms conducting early lactation preventive CT [74], but the underlying process could not be elucidated. For better understanding of the impact of timing of preventive CT on lameness episodes, cows without previous lesion or lameness history need to be enrolled using a prospective longitudinal approach. This is because CT-related benefits might be masked by events occurring before trimming. For instance, cows with horn lesion history had increased bone growth and exostosis around the pedal bone [76]. Since multiparous cows are more likely to be lame and in a case of CHDL history, these factors may reduce the efficacy of CT irrespective of the timing during lactation. 

Peer-reviewed papers on the frequency of preventive CT and lameness occurrence in dairy cows are scarce, with only a few studies demonstrating lower levels of CHDL in farms where cows underwent twice preventive CT during lactation compared to farms where the practice was once per lactation [18,35]. This might be linked to the aetiopathogenesis of CHDL as repeated CT might improve the stability of pedal bone and reduce the tendency of relapse of previous lesions. Nevertheless, the need for more preventive trimming during lactation could be replaced by identifying the appropriate timing and CT technique that results in greater reduction in horn lesions under various management systems. 

The application of computerized CT database programs to record the claw health data routinely at each claw CT visit provides the basis for monitoring hoof health and adjusting lameness management practices [77]. These database programs allow for instant recording of claw lesions during trimming and treatment, immediate analysis upon completing CT, and prompt access at any future time point [77,78]. The comprehensive claw health data consist of records on claw lesions, their severity scores, and exact location [79,80], as well as cow and farm level scores useable for comparing claw data at subsequent visits [77]. These features have the potential of curtailing some of the problems associated with assessing the benefits of CT in dairy herds. For instance, computerized CT databases are in the best position to have comprehensive records of lameness and lesions history of dairy cows. Researchers can easily enroll cows based on certain inclusion criteria to elucidate the efficacy of CT in lameness management. Recently, large-scale collection of computerized claw data has been employed for estimating genetic heritabilities of claw lesions [81,82]. Other CT database programs are integrated with performance variables including milk yield and reproduction data [77], and these variables are influenced by routine CT in dairy herds [83,84]. Thus, not only does electronic recording of claw health data provides a better and accurate approach when investigating the impact of CT on production, it also makes available the selection and analysis of production groups. In addition to the information provided in Figure 1, studies reporting associations between CT and claw health in dairy cows are presented in Table 1. Although most of the studies described the occurrence of clinical lameness and claw lesions, only few of them applied computerized CT database programs [42,75].

## 6. Association between CT and Behavioral Variables

The freedom to express normal behavior is essential in assessing the welfare of the dairy cow. Since the aim of CT is to improve claw health and well-being, the behavioral changes associated with lameness should be minimal or short-lived after the procedure. Post-CT behavioral alterations included significant increase in lying duration and reduced walking speed [3,48,83] increased jaw movements and eating time [3] and decreased neck activity [84]. Depending on study duration and observation points, CT-related behavioral changes were observed for hours to weeks post-CT [3,83]. These findings indicated that CT induced stress and discomfort in dairy cows, which was followed by behavioral responses to annul the effect.

As shown in Table 2, several studies have attempted to investigate the associations between corrective CT and behavior in dairy cows affected with specific claw lesions [45,83,84]. The findings can be divided into those showing the short- [5,24,45,84] and long-term impact [85]. For the purpose of this review, short term refers to observational period ranging from days to 2 weeks post CT, whereas study duration greater than 2 weeks to 1 complete lactation (305 DIM) was considered as long-term. This was based on the criteria used in categorizing acute and chronic lameness cases in previous studies [20,45]. Acutely lame cases were considered in most of the studies investigating the short-term outcomes, whereas Pavlenko et al. [85] and Cruz et al. [86] might have enrolled chronically lame cows. Corrective CT led to significant reduction in LS, while increasing the number of steps/day, pressure nociceptive threshold (PNT) and lying down time in cows affected with CHDL compared to those with DD [5,83,87]. The results indicated recovery from acute CHDL-related lameness episodes, whereas the lower impact of CT on behavioral parameters in DD-affected cows highlighted the need for other management plan relating to its infectious nature.

Corrective CT for specific claw lesions is also associated with immediate painful sensation which may affect a cow’s behavior. For instance, significantly higher leg movements and reduced lying down time (for 3 weeks) were reported in cows following corrective CT for SU and WLD [24,85]. These findings suggest that CT may affect cows’ behavior differently depending on the lesion and the severity level, as well the importance of pain management in annulling the immediate effects [87]. Nevertheless, most of the highlighted studies were lacking a detailed description of the claw health of the enrolled animals. This limits ascribing the various behavioral changes either to CT or lameness alone. 

## 7. Association between CT and Physiological Parameters

Lameness causes pain and stress in affected animals [5,88]. Significant increase in clinical and physiological parameters such as heart rate and variability [88], plasma cortisol and haptoglobin levels [6,24] have been demonstrated in lame dairy cows, thus, showing lameness-associated stress. Likewise, corrective CT is associated with changes in physiological parameters in dairy cows. Clinical parameters such as heart rate and respiratory rate increased significantly in trimmed cows compared to controls [25,89]. However, these clinical parameters may not provide the real picture of the ongoing stress in lame cows. A stronger indication of stress-related changes included significant increase in the concentrations of blood cortisol, glucose, and fecal cortisol metabolites in lame cows compared with sound cows after corrective CT [24,90].

Claw temperatures of the trimmed foot have also been demonstrated as a measure of physiological changes associated with CT [5,92]. Corrective and functional CT led to a decrease in claw temperature of the feet affected with CHDL [4] and infectious claw lesions [92], respectively. Although an initial increase in claw temperature was observed following the use of an electric grinder in leveling the claws, both studies attributed such temperature reductions to the positive effect of CT in correcting claw dimensions and weight bearing. To make valid inference about the impact of CT on physiological parameters, it is pertinent to determine if alterations in stress indicators result from actual horn removal during trimming or cow restraint. Such deduction is limited in studies where either lame cows were enrolled [24,89,90] or claw health was not considered [89,91]. This could be clarified by investigating the physiological parameters in non-lame cows following functional and sham trimming.

Overall, comparisons between the reviewed studies need to consider the research designs, claw health status of the animals, and applied CT methods. There are more data regarding the impact of corrective CT (treatment of claw lesions) on behavioral and physiological parameters in dairy cows (Figure 2). More research is needed to understand how these parameters are affected by preventive CT (horn removal process only). To achieve this, comparisons of behavioral and physiological measures need to be done without the inclusion of lame cows. Presently, there is no peer reviewed paper on behavioral changes associated with preventive CT in non-lame cows, whereas our search revealed only two studies to have reported changes in some physiological parameters in non-lame cows after preventive CT [26,91]. However, both studies are limited due to small sample size and undetailed description of claw health and CT methods. Of all the reviewed papers that investigated the relationship between CT and behavioral or physiological parameters, only six studies provided a detailed description of the claw health status and applied CT technique (Table 2). 

## 8. Association between CT and Production Variables

Milk yield, reproduction performance and production lifespan are important measures of production in dairy cows. Literature findings relating to the association between CT and milk yield can be categorized based on the short-term [10,22,90,93] and long-term outcomes [17,70,85,94,95]. Milk yield decreased significantly in the short term after prophylactic or corrective CT [45,84,90,95]. These results may be connected to the stress-related changes in dairy cows following the CT procedure, especially in the absence of pain management [24]. In contrast, Ando et al. [94] and Kibar and Cabalayan [95] reported significant increases in long-term milk yield after CT. These latter studies were conducted in lame cows and the positive impact on milk yield could be associated with improved locomotion score and behavioral activity linked to production, as well as physiological response to alleviate stress in the affected animals [20]. The reviewed studies suggest that CT augments the cows’ capacity to maintain milk yield during lactation by reducing the incidence of lameness episodes. In other studies, corrective CT had no effect on milk yield in dairy cows [10,17,23,70,94]. The inconsistencies in these studies were attributed to lower severity of claw lesions observed during CT [10], influence of different management and housing designs in the studied farms [17], and the efficacy of the applied CT method in reducing claw lesions incidence during the observation period [17,70]. 

The studies reporting short-term decrease in milk yield following CT highlight the need to clarify if such changes result from cow restraint or actual horn removal. During cow restraint, the interruption of cows’ daily activity relating to feeding and lying behavior could influence milk production. In the long term, alterations in milk yield attributed to CT need to be differentiated from the impact of the existing claw lesion. This can be better understood by conducting a similar research in non-lame cows consisting of the actual trimmed and sham trimmed groups. Another area requiring more research is the role of different CT methods (prophylactic CT) on milk yield and other production variables in non-lame cows. The two studies that considered the association between CT and reproductive parameters found no significant difference in conception rate before and after corrective CT [70], and between trimmed and control groups [94]. These studies are limited based on undetailed description of claw health of the enrolled animals and the applied CT method. Machado et al. [71] focused on the influence of body condition score and claw horn lesions at dry-off on reproductive performance and survivability in dairy cows. Although the cows were hoof-trimmed before dry-off, the influence of CT on the outcome variables was not considered. (Table 3).

## 9. Other Considerations Regarding CT

Aside from the reviewed sections, other considerations for routine CT include housing design, management, and expertise of the hoof trimmer. The growth rate and wear of the claw, as well as kinetic effects differ between various stall designs with floor types being a significant factor [79]. Recovery rate determined by the onset of sound locomotion score was significantly higher in treated cows housed in tie stall barns compared to those in free stall barns [56]; thus, indicating the influence of management systems. The importance of expertise in CT has been reported in a few studies. These studies emphasized that only correct and adequate CT is beneficial for claw health [32,59]. Findings such as high degree of imbalance (based on the minimum sole thickness) in majority of the cows’ feet trimmed before slaughter [96], and significantly lower recovery rates among lame cows trimmed by the farmer compared to those trimmed by professional hoof trimmers [97,98] indicated that faulty CT may exacerbate claw lesions.

## 10. Conclusions

The scientific literature advocates that CT is beneficial for lameness management, as well as improving the welfare and production of dairy cows. There are several studies relating to the association between CT, welfare and production measures; however, determining the actual role of CT on these areas is faced with constraints such as undetailed descriptions of the claw health of enrolled animals and applied CT techniques. Different CT methods are currently used by hoof trimmers, but the benefits of their usage under various management systems or claw wear conditions need to be ascertained. The areas requiring more research regarding preventive CT include timing, frequency, long-term protective effect and identifying the cows that will benefit more from such an intervention during lactation. Such information will be vital in educating farmers on making on-farm decisions regarding CT as a lameness management strategy.

## Figures and Tables

**Table 1:** Reviewed studies reporting associations between claw trimming and claw health in dairy cows and those with detailed description of applied claw trimming (CT) technique.

Categories Based on Studied Variables and Description of CT	Parameters Used	References
Claw traits and gait parameters	Claw dimensions, weight distribution, pressure on various claw regions, symmetry between the claws	Archer et al. [9], Carvalho et al. [11], van der Tol et al. [16], Gomez et al. [21], Chapinal et al. [25], Ouweitjes et al. [28], Meyer et al. [30], Nuss and Paulus [31], Tanida et al. [33], Thorup et al. [46], Manske et al. [55], Armbretch et al. [69]
Clinical lameness	Lameness recovery rate, locomotion score, lameness prevalence and incidence	Passos et al. [5], Groenvelt et al. [8], García-Muñoz et al. [10], Mahendran et al. [17], Hernandez et al. [19], Thomas et al. [20], Ouweitjes et al. [28], Thomas et al. [42], Bryan et al. [43], Leach et al. [44], Montgomery et al. [45], Thorup et al. [46], Miguel-Pacheco et al. [48], Schulz et al. [47], Daros et al. [75], Van Hertem et al. [84]
Claw lesions	Claw lesions as outcomes or described	Passos et al. [5], Groenvelt et al. [8], van der Tol et al. [16], Thomas et al. [20], García-Muñoz et al. [10], Mahendran et al. [17], Manske et al. [18], Ouweitjes et al. [28], Thomas et al. [42], Somers et al. [53], Leach et al. [44], Montgomery et al. [45], Thorup et al. [46], Schulz et al. [47], Miguel-Pacheco et al. [48], Thomsen et al. [49], Manske et al. [55], Armbretch et al. [69], Van Hertem et al. [84]
Description of applied CT	Step by step description of the CT	Thomas et al. [20], Manske et al. [18], Nuss and Paulus [21], Ouweitjes et al. [28], Bryan et al. [40], Thomas et al. [42], Montgomery et al. [45], Van Hertem et al. [84]

**Table 2:** Reviewed studies reporting associations between claw trimming and physiological and behavioral parameters in dairy cows and specific studies with detailed description of claw health and applied CT technique.

Categories of Physiological and Behavioral Variables	Parameters	References
Activities	Lying down (time and bouts), number of steps, neck activity, rumination, leg movements	Weigele et al. [3], Passos et al. [5], Janßen et al. [24], Chapinal et al. [25], Montgomery et al. [45], Miguel-Pacheco et al. [48], Chapinal et al. [83], Van Hertem et al. [84], Pavlenko et al. [85], Cruz et al. [86]
Stress-related indicators (enzymes and blood constituents)	Fecal Cortisol, serum cortisol, glucose, lactate, Pressure nociceptive threshold,	Passos et al. [5], Janßen et al. [24], Korkmaz et al. [26], Kovacs et al. [88], Rizk et al. [89], Pesenhofer et al. [90], Nishimori et al. [91]
Clinical parameters	Respiratory rate, heart rate, rectal temperature, claw temperature	Passos et al. [5], Korkmaz et al. [26], Kovacs et al. [88], Alsaaod et al. [92]
Studies with detailed description of claw health and applied CT technique		Weigele et al. [3], Janßen et al. [24], Montgomery et al. [45], Miguel-Pacheco et al. [48], Van Hertem et al. [84], Pesenhofer et al. (90)

**Table 3:** Reviewed studies reporting associations between claw trimming and production variables in dairy cows and specific studies with detailed description of claw health and applied CT technique.

Categories of Production Variables	Parameters	References
Milk yield	Milk yield (L/kg) in the short-term (days to 2 weeks after CT)	García-Muñoz et al. [10], Erol et al. [23], Gomaa et al. [76]; Pesenhofer et al. [90], Nishimori et al. [91]
	Milk yield in the long term (months and 1 lactation after CT)	Mahendran et al. [17], Erol et al. [23], Maxwell et al. [70], Machado et al. [71], Pavlenko et al. [85], Kibar and Çağlayan [95]
Reproductive performance	Conception rate	Machado et al. [71], Maxwell et al. [70], Ando et al. [94]
Culling risk or production lifespan	Culling risk/death	Machado et al. [71]
Detailed description of claw health and applied CT technique		Maxwell et al. [70], Pesenhofer et al. [90]
